# Steroid-Responsive Seronegative Immune-Mediated Necrotizing Myopathy Likely Triggered by Coxsackie B Virus: A Case Report

**DOI:** 10.7759/cureus.63394

**Published:** 2024-06-28

**Authors:** Mukesh Kumar, Syed M Ali, Vinod Kumar, Ahmad Alsibai, Om Parkash

**Affiliations:** 1 Neurology, Tower Health Medical Group, West Reading, USA; 2 Hospital Medicine, Cleveland Clinic Abu Dhabi, Abu Dhabi, ARE; 3 Internal Medicine, Tower Health Medical Group, West Reading, USA

**Keywords:** creatine kinase, steroids, immune-mediated inflammatory myopathy, alcoholic cirrhosis, coxsackie b virus myositis

## Abstract

Viral myositis can be mistaken for other types of myopathies, and the main causes of muscle damage are direct myotoxic effect and immune-mediated mechanisms. The biochemical parameters, electromyography (EMG), and muscle biopsy findings can be similar in viral myositis and idiopathic inflammatory myopathies. Viruses are rarely isolated from muscle biopsy specimens, so clinical evaluation and ancillary tests are necessary for a definitive diagnosis. Viral etiology is suspected when weakness occurs after a respiratory or gastrointestinal infection. Coxsackieviruses, particularly A9 and B5, can cause myositis and muscle necrosis. This is a case of a 47-year-old female with a history of alcoholic cirrhosis and a recent coxsackie B virus infection presented with weakness, numbness, and body pain. Creatine kinase levels were elevated but tests for extended myositis panel and antibodies were negative. A muscle biopsy revealed immune-mediated inflammatory myopathy. After a week without improvement, the patient received IV methylprednisolone followed by prednisone taper leading to improvement in symptoms. Prolonged myalgia has been observed in patients recovering from coxsackie A infections. The role of coxsackie B in causing myositis is still disputed and requires more reported data and guidelines. Clinicians should consider testing for coxsackie B as a potential cause of weakness. Awareness of potential complications like myositis can aid in effective patient management. More cases are needed to determine the significance of steroid use in managing coxsackie B-related muscle weakness.

## Introduction

Immune-mediated necrotizing myopathy (IMNM) is a form of myositis distinguished by severe muscle weakness, significantly elevated creatine kinase (CK) levels, and muscle necrosis with minimal inflammation observed in muscle biopsy [1]. Myositis is a serious and uncommon complication of coxsackie B virus infection [2]. In this case report, we present the case of a 47-year-old patient who experienced weakness in both upper and lower extremities, diffuse body pain, and motor deficit in the lower extremities following an infection with Group B coxsackie virus. The patient also had a history of decompensated alcoholic cirrhosis. The CK blood level increased to 1678 U/L. The inflammatory workup was negative for extended myositis panel and HMG CoA (β-hydroxy β-methylglutaryl-CoA) reductase antibodies, and muscle biopsy revealed immune-mediated inflammatory myopathy with necrotizing features, with the absence of any antibodies. Due to the absence of spontaneous recovery for approximately one week, the patient was subsequently treated with a trial of intravenous methylprednisolone 1 g for five days, which led to improvement in all clinical and paraclinical abnormalities.

## Case presentation

A 47-year-old female with a medical history of alcohol abuse, alcoholic cirrhosis (Child-Pugh class C) with ascites, and depression presented to the hospital with complaints of generalized weakness, numbness, and diffuse body pain for the past four days. The patient was recently discharged from the hospital after being admitted for the management of hepatitis and decompensated alcoholic cirrhosis (MELD-Na 25) with ascites. She was discharged on a prednisone taper dosing regimen (40 mg to 10 mg for one month), along with spironolactone 50 mg and furosemide 20 mg daily. The patient completed the course of steroids. The patient reported that her symptoms began with numbness in her right leg, which she initially attributed to a pinched nerve. Over the next two days, she experienced severe pain in both shoulders and inner thighs (rated 9-10/10 intensity) and generalized weakness, rendering her unable to walk. On evaluation, the patient was hemodynamically stable. Routine laboratory findings are shown in Table 1. On neurological examination, the patient was alert and oriented to time, person, and place. Cranial nerves 2-12 were intact, and motor examination revealed normal bulk and tone. Strength was 4/5 for shoulder abduction bilaterally and 4+/5 for elbow flexion and extension. Lower extremity strength was 2/5 for hip flexion bilaterally, -4/5 for hip abduction bilaterally, and 5/5 for knee flexion, knee extension, ankle flexion/dorsiflexion, and ankle eversion/inversion. The patient's activities were significantly limited by tenderness in both upper and lower extremities. Sensory examination showed diminished sensation to cold temperature in a length-dependent pattern in the lower extremities, with intact proprioception and decreased vibratory sensation in the toes bilaterally. Reflexes were 2+ throughout, and the Babinski sign was negative. The finger-to-nose testing did not reveal any dysmetria in the cerebellar exam.

**Table 1:** Routine laboratory tests

Blood Test	Result	Reference Range (Units)
White Blood Cells (WBCs)	17.3	4.8-10.8 x 10^9^/L
Hemoglobin (Hb)	10.9	12.0-16.0 g/dL
C-Reactive Protein (CRP)	6.1	<1.00 mg/L
Sodium	135	136-145 mmol/L
Potassium	3.6	3.5-5.1 mmol/L
Creatinine	0.56	0.6-1.3 mg/dL
Erythrocyte Sedimentation Rate (ESR)	59	0-20 mm/h
Creatine Kinase (CK)	1678	<200 U/L
Aspartate Aminotransferase (AST)	277	13-39 U/L
Alanine Aminotransferase (ALT)	92	7-52 U/L
Alkaline Phosphatase (ALP)	322	34-104 U/L
Ammonia	106	16-53 µmol/L
Calcium	7.7	8.6-10.3 mg/dL
Vitamin B12	1500	180-914 pg/mL
Thyroid-Stimulating Hormone (TSH)	2.5	0.4-5.3 mIU/L

The brain MRI indicated mild diffused atrophy with no additional abnormalities. The cervical spine MRI revealed multilevel degenerative disc disease at C5-C6, C6, and C7, but no acute pathology. Infectious workups, including influenza panel, COVID, treponemal pallidum antibody, blood cultures, and ascitic fluid cultures were negative. Special tests including autoimmune panel and Lyme antibodies were performed and are detailed in Table 2. 

**Table 2:** Autoimmune and infection serology HMG CoA, β-hydroxy β-methylglutaryl-CoA.

Blood Test	Result	Reference Range (Units)
Viral Hepatitis Panel	Negative	Negative
Extended Myositis Panel	Negative	Negative
HMG CoA Reductase Antibodies	Negative	Negative
Serum Protein Electrophoresis (SPEP)	Negative	Negative
Kappa/Lambda ratio	1.88	0.2-1.65
Serum Copper	71 µg/dL	70-175 µg/dL
Ceruloplasmin	21 mg/dL	18-53 mg/dL
Antinuclear Antibody (ANA)	Negative	Negative
Antineutrophil Cytoplasmic Antibodies (ANCA)	Negative	Negative
Lyme Antibodies	Negative	Negative
Anti-Smooth Muscle Antibody	Negative	Negative
Antimitochondrial Antibody	Negative	Negative
Anti-Ribonucleoprotein (RNP) Antibody	Negative	Negative
Anti-SSA/SSB Antibodies	Negative	Negative
Rheumatoid Factor	21 IU/mL (Mildly Positive)	<14 IU/mL

Coxsackie panel resulted in high titers positive for B3 and B5 as shown in Table 3.

**Table 3:** Coxsackie virus panel

Blood Test	Result	Reference Range (Units)
Coxsackie Viral Panel	1:64 (High Titers for Coxsackie B3 and B5)	<1:8 (Negative)

**Figure 1:**
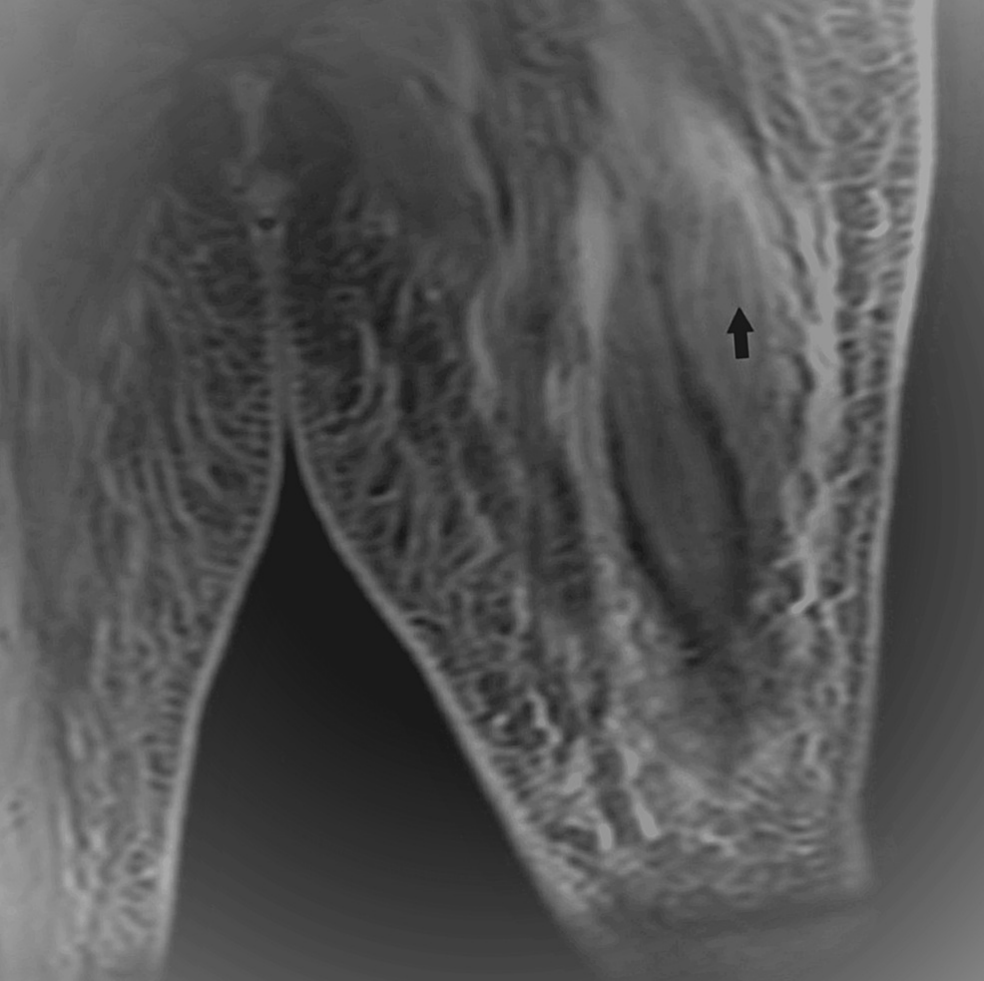
MRI left thigh STIR sequence MRI, magnetic resonance imaging; STIR, short tau inversion recovery.

The CT chest revealed bilateral pleural effusion and a large amount of ascites. The CT (computed tomography) images of the abdomen and pelvis did not show any evidence of malignancy. The MRI (magnetic resonance imaging) images of the left thigh showed significant diffuse inflammatory changes in the deep and subcutaneous tissue, along with a large fluid collection associated with abnormal enhancement (Figure 1). A muscle biopsy was performed, which showed features of immune-mediated inflammatory myopathy including myopathic inflammatory features characterized by degenerating/regenerating fibers and rare areas of endomysial lymphocytic and histiocytic infiltration with focal myophagocytosis. HLA and complement upregulation was seen in the necrotic and degenerating muscle fibers. Esterase and acid phosphatase positivity was also noted in the necrotic fibers. These features support the diagnosis of an IMNM. Antibody tests were negative. The patient was then treated with intravenous methylprednisolone 1 g for five days, which resulted in an improvement in strength, C-reactive protein, and CK levels. The patient was started on a prednisone taper dosing starting from 60 mg and discharged for rehabilitation.

## Discussion

Viral myositis is often mistaken for idiopathic "polymyositis" or dermatomyositis. Severe cases of viral myositis with extensive necrosis can mimic necrotizing myopathy and drug-induced myopathy [2]. The mechanisms of viral infection include direct cytopathic/cytolytic effects, molecular mimicry, immune complex formation, and immune dysregulation. However, direct myotoxic effect and immune-mediated mechanisms are the main causes of muscle damage [3]. Spontaneous recovery usually occurs, except in cases where complications such as rhabdomyolysis, myoglobinuria, acute renal failure, cardiac arrhythmias, and compartment syndrome develop. The common viruses that cause rhabdomyolysis include influenza, enteroviruses (coxsackie A and B), Epstein-Barr virus, human immunodeficiency virus (HIV), and herpes simplex virus [4]. Biochemical parameters, electromyography (EMG), and muscle biopsy findings may be similar in both viral myositis and idiopathic inflammatory myopathies. Muscle biopsy specimens rarely yield isolated viruses. A definitive diagnosis requires clinical evaluation and corroborative evidence from ancillary tests. However, when weakness develops acutely or sub-acutely following a respiratory or gastrointestinal infection (such as hepatitis in our patient), suspicion of a viral cause increases. Idiopathic inflammatory myopathy can be caused by enteroviruses, particularly coxsackie viruses A9 and B5, leading to myositis and muscle necrosis [5]. Coxsackie viruses are non-enveloped viruses with linear single-stranded RNA. Group A coxsackie viruses cause flaccid paralysis due to generalized myositis, while group B coxsackie viruses cause spastic paralysis due to neuronal tissue degeneration and focal muscle injury [6]. Group B coxsackie viruses also infect the heart, pleura, pancreas, and liver, causing pleurodynia, myocarditis, pericarditis, and hepatitis. Coxsackie viruses are known for their myotropism, including their association with Bornholm disease or epidemic myalgia [7]. Clinical evidence of this type of myopathy may be accompanied by elevated levels of creatinine kinase and myoglobin. Although chronic alcoholic use disorder and hepatic cirrhosis can cause myopathy, the acute presentation and abstinence from alcohol make these causes less likely in our patient. Initially treated for alcoholic hepatitis and sent home on a steroid taper, the patient returned after one month with severe muscle weakness. A viral panel revealed higher titers for coxsackie B, particularly B3 and B5. Most infections are asymptomatic, and the factors contributing to individual susceptibility to the virus remain largely unknown [8]. Possible factors include previous immune status, immune deficiency restricted to certain strains, and higher pathogenic potency of mutants. It is possible that the initial hepatitis in this patient was caused by the coxsackie virus, and the use of steroids during an active infection led to complications of coxsackie B myositis. Ultimately, the improvement in muscle strength was attributed to the use of steroids. However, it is unclear whether the steroids helped improve strength or if the resolution of the infection itself contributed to the improvement. This emphasizes the importance of repeat coxsackie panel testing. Prolonged myalgia during convalescence from coxsackie A infections has been described in patients for several decades. The involvement of coxsackie B in causing myositis is a topic of ongoing debate in the literature, and additional case reports are needed to further explore this relationship. This particular case highlights the significance of coxsackie B myositis, specifically B3 and B5 strains, which are rare causes of focal weakness and have not been extensively documented in the literature. When evaluating patients with weakness and myalgias, especially those with chronic conditions like alcoholic liver cirrhosis, it is crucial to obtain a comprehensive medical history to identify any prodromal symptoms. Clinicians should consider expanding serological testing for the coxsackie panel as a potential cause of weakness, regardless of its impact on the treatment plan. Being aware of potential complications such as myositis can help guide clinicians in effectively managing patients and save time in similar situations. It is essential to report more cases of muscle weakness caused by coxsackie B and investigate the potential relevance of steroid use in its management. 

## Conclusions

This case illustrates the potential association of IMNM with coxsackie B infection. There have not been clear guidelines or literature available to link this association. It is very important to take a comprehensive history to identify prodromal symptoms when working up weakness and myalgias, especially in patients with chronic problems like alcoholic liver cirrhosis. Clinicians should broaden serological testing for coxsackie panel as a potential cause of weakness. Regardless of its influence on the treatment plan, awareness of potential complications like myositis will help direct clinicians to manage patients effectively and save time in similar situations. More cases need to be reported in patients who have muscle weakness due to coxsackie B and to determine whether the use of steroids has any importance in management.
